# The Therapeutic Potential of Psilocybin

**DOI:** 10.3390/molecules26102948

**Published:** 2021-05-15

**Authors:** Henry Lowe, Ngeh Toyang, Blair Steele, Henkel Valentine, Justin Grant, Amza Ali, Wilfred Ngwa, Lorenzo Gordon

**Affiliations:** 1 Biotech R & D Institute, University of the West Indies, Mona 99999, Jamaica; lowebiotech@gmail.com (H.L.); henkelval@yahoo.com (H.V.); justin@psyence.com (J.G.); amza@psyence.com (A.A.); 2 Vilotos Pharmaceuticals Inc., Baltimore, MD 21202, USA; ngeh.toyang@flavocure.com; 3 Flavocure Biotech Inc., Baltimore, MD 21202, USA; 4 Institute of Human Virology (IHV), University of Maryland School of Medicine, Baltimore, MD 21202, USA; 5 The Psyence Group, Toronto, ON M5J 2J1, Canada; 6 Brigham and Women’s Hospital, Dana-Farber Cancer Institute, Harvard Medical School, Boston, MA 02215, USA; wngwa@bwh.harvard.edu; 7 Caribbean School of Medical Sciences, Kingston 99999, Jamaica; lorenzogordon2011@yahoo.com

**Keywords:** magic mushrooms, psilocybin, psychedelic, neuropharmaceuticals, neurotherapeutics, addiction, anxiety, depression, cancer, psychopharmacology

## Abstract

The psychedelic effects of some plants and fungi have been known and deliberately exploited by humans for thousands of years. Fungi, particularly mushrooms, are the principal source of naturally occurring psychedelics. The mushroom extract, psilocybin has historically been used as a psychedelic agent for religious and spiritual ceremonies, as well as a therapeutic option for neuropsychiatric conditions. Psychedelic use was largely associated with the “hippie” counterculture movement, which, in turn, resulted in a growing, and still lingering, negative stigmatization for psychedelics. As a result, in 1970, the U.S. government rescheduled psychedelics as Schedule 1 drugs, ultimately ending scientific research on psychedelics. This prohibition on psychedelic drug research significantly delayed advances in medical knowledge on the therapeutic uses of agents such as psilocybin. A 2004 pilot study from the University of California, Los Angeles, exploring the potential of psilocybin treatment in patients with advanced-stage cancer managed to reignite interest and significantly renewed efforts in psilocybin research, heralding a new age in exploration for psychedelic therapy. Since then, significant advances have been made in characterizing the chemical properties of psilocybin as well as its therapeutic uses. This review will explore the potential of psilocybin in the treatment of neuropsychiatry-related conditions, examining recent advances as well as current research. This is not a systematic review.

## 1. Introduction

The word “psychedelic” (*psyche* (i.e., the mind or soul) and *delos* (i.e., to show)) has Greek origin and was first coined by psychiatrist Humphry Osmond in 1956 [1,2], who had been conducting research on lysergic acid diethylamide (LSD) at the time. Psychedelics are a class of hallucinogenic drugs (“hallucinogens”) that produce mind-altering and reality-distorting effects, known as hallucinations, once ingested. Hallucinations typically trigger delusions, emotional swings, feelings of detachment and derealization. Hallucinogens are generally classified into two main categories, (1) dissociative drugs, such as dextromethorphan (DXM), ketamine *Salvia divinorum* and Phencyclidine (PCP) [3,4] and (2) classic serotonergic and dopaminergic hallucinogens that interact with serotonin and dopamine receptors, respectively.

Classes of classic serotonergic and dopaminergic hallucinogens include (1) Lysergamides (amides of lysergic acid)—LSD/LAD, and ergotamine, (2) Phenethylamines such as MDMA (ecstasy), MDMA-like drugs such as *p*-methoxy methamphetamine (PMMA), mescaline and mescaline-derived compounds like TMA, DOM, DOET, DOI (2,5-dimethoxy-4-iodoamphetamine), and DOC (2,5-dimethoxy-4-chloroamphetamine), and (3) **Tryptamines** such as N,N-dimethyltryptamine (DMT) and its derivatives alpha-methyltryptamine (AMT), 5-methoxy-N,N-dimethyltryptamine (5-MeO-DMT) and 5-methoxy-N,N-diisopropyltryptamine (5-MeO-DIPT), and psilocin (4-hydroxy-N,N-dimethyltryptamine also known as 4-OH-DMT) and psilocybin ([3-(2-Dimethylaminoethyl)-1*H*-indol-4-yl] dihydrogen phosphate. In addition to psilocin, other metabolites of psilocybin include; (1) 4-hydroxyindole-3-yl-acetaldehyde (4H1A), (2) 4-hydroxyindole-3-yl-acetic-acid (41-IIAA), and (3) 4-hydroxytryptophol (41-IT) [5]. On the same tangent, psilocybin, lysergic acid diethylamide, and lysergic acid amide are classified as indoleamine hallucinogens [6].

**Figure 1:**
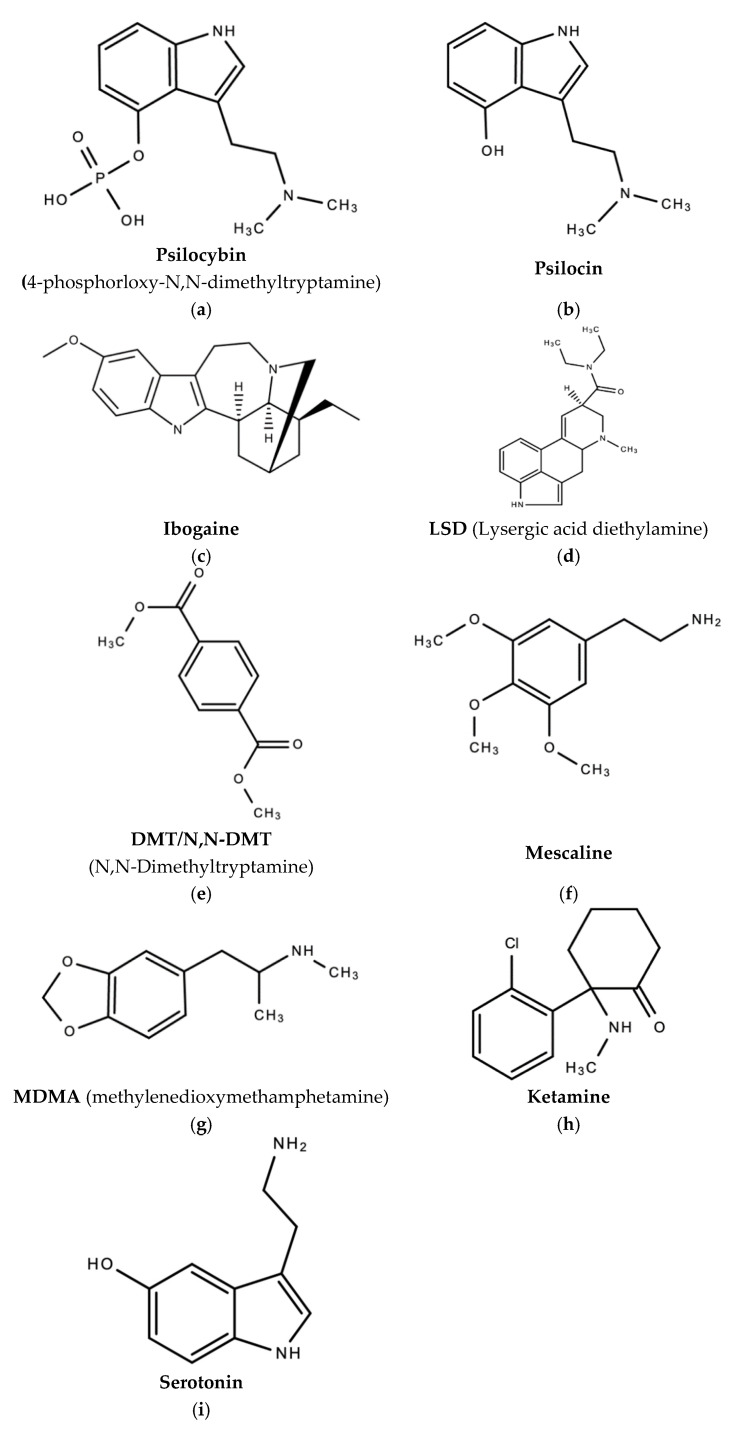
Chemical structures of classic serotonergic psychedelic compounds (**a**–**h**) and the neurotransmitter serotonin (**i**). These substances all utilize the 5-hydroxytryptamine_2A_ receptor.

Classic psychedelic (serotonergic) drugs interact with the serotonin receptors (5-HT/5-hydroxytryptamine receptors) and their subtypes densely located within the brain [7,8,9]. These receptors mediate emotions and moods such as anxiety and aggression, cognition, sex, learning memory, appetite along with other biological, neurological and neuropsychiatric processes [8,10]. These 5-HT receptors are also located in the central and peripheral nervous systems [11,12]. Serotonin receptors are the target of multiple recreational and pharmaceutical drugs such as hallucinogens, empathogens, antipsychotics, antidepressants, antiemetics, antimigraine agents and anorectics [10]. Figure 1 shows the chemical structures of classic psychedelic drugs and the neurotransmitter serotonin.

Of all psychedelic drugs, psilocybin is reported to have the most favorable safety profile [13]. Despite the lack of studies investigating the comparative efficacies of psilocybin and psychedelic drugs for the treatment of mood and anxiety disorders, the vast evidence-based data that exist for psilocybin alone suggest that psilocybin may be the most efficacious psychedelic drug for treating such disorders.

In a 2017 Global Drug Survey, it is estimated that approximately 20.6% of people worldwide who used drugs of any type, selectively used magic mushrooms within their lifetime up to that year [14]. This review will focus on psilocybin, the main psychoactive component of magic mushrooms (psilocybin-producing mushrooms), that has been utilized for thousands of years in mushroom-worshiping ceremonies in old-world cultures [15,16]. In addition to the known recreational, spiritual, and religious uses of magic mushrooms, there is significant medicinal value, as evidenced by anecdotal reports and scientific studies. Table 1 below lists the diseased states in which psilocybin-assisted therapy is being explored.

Mental health and substance-use disorders such as depression, anxiety-related disorders, bipolar disorder, autism, psychoses such as schizophrenia, and substance-dependence disorders are of significant global burden [17,18]. Up to 2020, it is estimated that 1 billion people may be affected by a mental health or substance disorder [19]. Anxiety-related disorders were the most burdensome mental health disorders in 2018, affecting an estimated 284 million people [20], while depression, the second-most common—affected an estimated 264 million people globally the same year [19,20]. Additionally, in 2018, alcohol-use disorder affected an estimated 107 million people globally, while drug use disorder (excluding alcohol) affected 71 million people globally [20]. Suicidality, which also correlates to mental health, is also of global burden. Statistics show that an estimated 800,000 people commit suicide annually [21]. With the COVID-19 pandemic, risk factors for mental health and substance-use disorders are expected to be exacerbated, with evidence of increased rates of anxiety, depression and distress [22].

**Table 1:** The potential therapeutic window of psilocybin-assisted therapy, that is, diseased states in which psilocybin-assisted therapy is being explored.

	Diseased State/Condition	Reference
1.	Alcohol dependence	[23,24,25]
2.	Stimulant dependence	[25]
3.	Cocaine addiction	[26,27]
4.	Tobacco addiction	[25,28,29,30]
5.	Nicotine addiction	[26,29]
6.	Opioid addiction	[25]
7.	Cannabis dependence	[25]
8.	Anxiety disorders such as: Post-traumatic stress disorder (PTSD), Generalized anxiety disorder (GAD), Obsessive–compulsive disorder (OCD) Advanced-stage cancer-related anxiety Psychological distress associated with existential crisis of terminal disease Adjustment disorder with anxiety	
		[26]
		[26]
		[31,32]
		[33,34,35,36,37]
		[26]
		[26,38]
9.	Cancer-related depression	[33,34,35,36,37,39]
10.	Treatment-resistant depression	[40,41,42,43,44]
11.	Major Depressive Disorder	[45]
12.	Severe existential depression	[26,33,36]
13.	Suicidality (ideation and actual attempts)	[13,46]
14.	Cluster (“suicide”) headaches	[6,47]
15.	Chronic pain	[48,49,50]
16.	Intractable phantom pain	[51]
17.	Demoralization	[52]
18.	Demoralization in older, long-term AIDS survivor men (OLTAS)	[53]
19.	Dysfunctional social cognition	[54]
20.	Maladaptive narcissism	[55]
21.	Borderline Personality Disorder (BPD)	[56,57]
22.	Narcissistic Personality Disorder (NPD)	[58,59,60]
23.	Epilepsy	[61]
24.	Psychopathy	[54]
25.	Emotional dysregulation and violence against one’s partner	[62,63,64]
26.	Inflammation	[49]

With the increase in the rate of mental disorders globally, now exacerbated by COVID-19, psychedelic-assisted psychotherapies, particularly psilocybin-assisted psychotherapies, may alleviate some of the challenges that face conventional psychiatric medicine.

In addition to having the potential to treat mood and anxiety disorders, psilocybin has also demonstrated analgesic effects as evidenced by numerous clinical studies on the treatment of cluster (“suicide”) headaches [6,47], intractable phantom-limb pain (PLP) [51], and chronic pain [48]. One possible mechanism of action of this analgesic property is via interaction with nociceptive and antinociceptive pathways [48]. In some cases, psilocybin was comparable to or more efficacious than traditional medications such as opioid analgesics [6,50].

A study by Nkadimeng and colleagues report dose-dependent analgesic, antioxidant, and anti-inflammatory properties of a certain *Psilocybe natalensis* species of magic mushroom [49]. Lipopolysaccharide (LPS)-stimulated macrophage cells were treated with three different 24-h extracts (hot water, cold water and ethanol) of *Psilocybe natalensis* mushrooms [49]. Antioxidant effects of *Psilocybe natalensis* mushrooms were confirmed when all three extracts inhibited lipopolysaccharide-induced nitric oxide [49]. Anti-inflammatory effects of *Psilocybe natalensis* mushrooms were confirmed upon the inhibition of prostaglandin E_2_, and interleukin 1β cytokine [49].

### 1.1. History

Despite thousands of years of psychedelic use in religion and recreation, the earliest known written record of magic mushroom use in the *Florentine Codex* (a manuscript of ethno-graphical research of Mesoamerica, particularly of Mexico and the Aztecs, compiled between 1529 and 1579 [10,15,63,65]

Classic psychedelic compounds like psilocybin, mescaline (isolated from the peyote cactus in 1897 by Arthur Heffter) [66] and dimethyltryptamine have been used in religious ceremonies in indigenous societies in South and Central America for centuries [66,67,68,69,70,71].

Modern and rigorous scientific study of psychedelics began in 1938 when Albert Hoffman of Sandoz Laboratories (Basel, Switzerland) discovered/synthesized lysergic acid diethylamide (LAD/LSD)—translated from the German word “Lysergsäurediethylamid”) [2,65,71]. This may be considered the birth of molecular psychiatry [72] and the beginning of the “first psychedelic renaissance”. On record, this may also be the first discovery of an ergot alkaloid derivative with medical value. During this period of early clinical research on psychedelics, LSD was the most studied psychedelic [2].

Nine years later, in 1947, Sandoz began marketing and distributing LSD as a psychiatric drug for the treatment of neurosis [73], alcoholism [74,75,76,77,78,79,80,81], criminal behaviour [82,83,84], schizophrenia [85,86,87,88,89], and sexual perversions [90]. LSD-25 was also used to treat autism [91,92], and verbal behaviour [93].

In 1957, Hofmann received a sample of dried *Psilocybe mexicana* mushrooms from a mycologist in Huautla de Jiménez in Oaxaca, Mexico [65]. This could be considered the beginning of the “second psychedelic renaissance”. To identify and convince himself of the mushroom’s bioactivity, Hoffman used paper chromatography to separate the various components of whole extracts of mushrooms, by ingesting each separated fraction [65]. The active fraction was then chemically characterized, crystallized and called psilocybin [65,94]. Hofmann and colleagues subsequently elucidated the structure and synthesis of psilocybin in 1958 [5,94,95,96] and a minor component of the extract, psilocin, a dephosphorylated form of psilocybin [65]. In the 1960s, Sandoz Pharmaceuticals (Basel, Switzerland) distributed Indocybin^TM^, a psychotherapeutic drug, in pill form, containing 2 mg psilocybin [65]. This period also saw experimental therapeutics with psilocybin as a probe for brain function [2], psilocybin for recidivism [97] and with psilocybin as an entheogen in religious people (divinity students) [98].

From the 1960s and 1970s, recreational use of psychedelics became central to the “hippie”, counterculture in the United States, and this ultimately fueled the United States Drug Enforcement Agency to prohibit psychotropic substances such as LSD, DMT (*N*,*N*-Dimethyltryptamine), Psilocybin and Mescaline, and label them as Schedule 1 drugs under the Controlled Substances Act 1970 (USA). As a result of this historical association with the highly sensationalized “hippie”, counterculture at the time, there has been lingering stigmatization of such substances, which has, in turn, hindered scientific research and innovation in psychedelic therapeutics [13], until recently.

### 1.2. Transition to Modern-Day Clinical Studies

Traditionally, the pharmaceutical industry, in reflection of societal understanding and governmental acceptance, rejected psychedelic research for a long time. Only recently has psychedelic research slowly made its reintroduction back into the paradigm of modern science and many clinical studies confirm the potential of psilocybin-assisted therapy as a promising adjunct to psychotherapy [15,99].

Early clinical studies with hallucinogens report the use of LSD-25 to treat the typical intractable behaviour seen in early infantile autism and childhood schizophrenia [88,91,100]. By the 1960s, over 40,000 individuals took part in psychedelic research studies, albeit with less rigorous clinical standards [101].

In 2004, University of California, Los Angeles (UCLA) researchers began clinical trials on psilocybin for the treatment of pain, anxiety and depression in patients with advanced-stage cancer. This may be considered the “third psychedelic renaissance”. A 2006 publication from the John Hopkins University heralded a new age for psychedelic research, reigniting worldwide interest [102]. This led to the formation of the psychedelic research unit, and eventually, the Center for Psychedelic and Conscious Research (John Hopkins University) in 2006, which has since published over eighty peer-reviewed articles on psychedelic research [103]. In September of 2020, the John Hopkins University built the Center for Psychedelic and Consciousness research, the first of its kind. To date, over 27,000 scientific articles have been published on psychedelic drugs, with over 1000 particularly on psilocybin [103]. Currently, psilocybin is the most studied psychedelic [2,13].

Amidst this renewed interest in psychedelic research, pharmaceutical interest has also increased. In 2018 Compass Pathways Ltd. (London, UK) received U.S. Food and Drug Administration (USFDA) approval of “breakthrough therapy” status for a psilocybin treatment they developed for treatment-resistant depression [104]. In the same year, the USFDA also approved SPRAVATO^®^, a ketamine analog developed by Johnson and Johnson for use in patients suffering from treatment-resistant depression [105]. In 2019, Usona Institute received USFDA “breakthrough therapy” status for a psilocybin treatment for major depressive disorder (MDD) [106].

**Figure 2:**
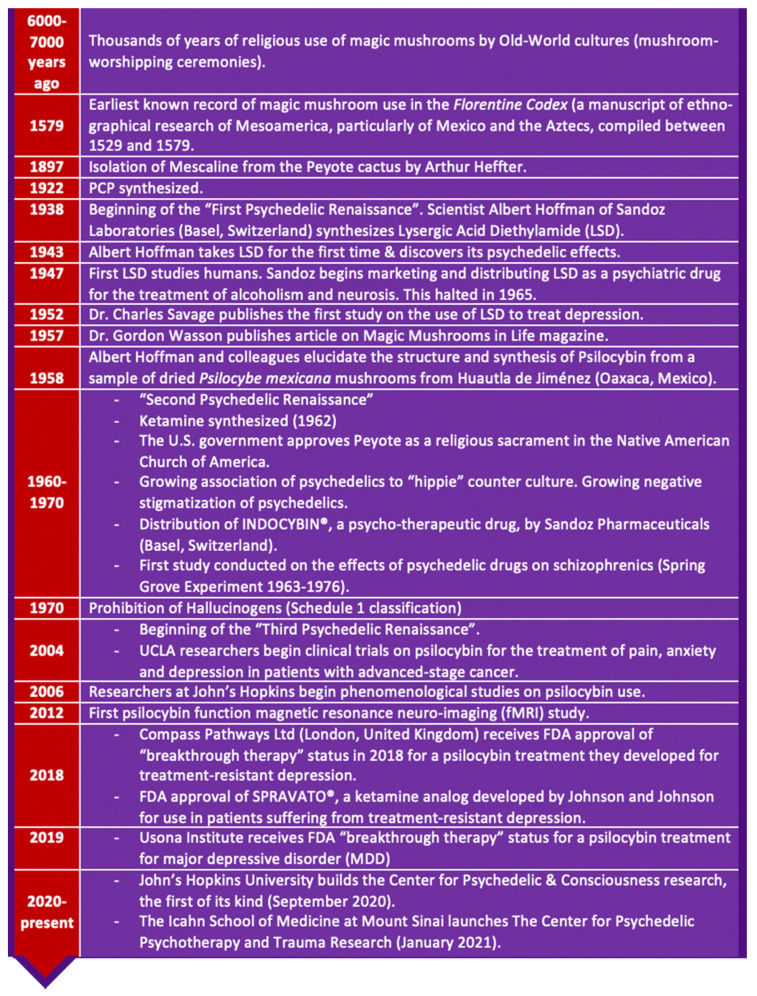
Historical timeline of psychedelic substances.

**Figure 3:**
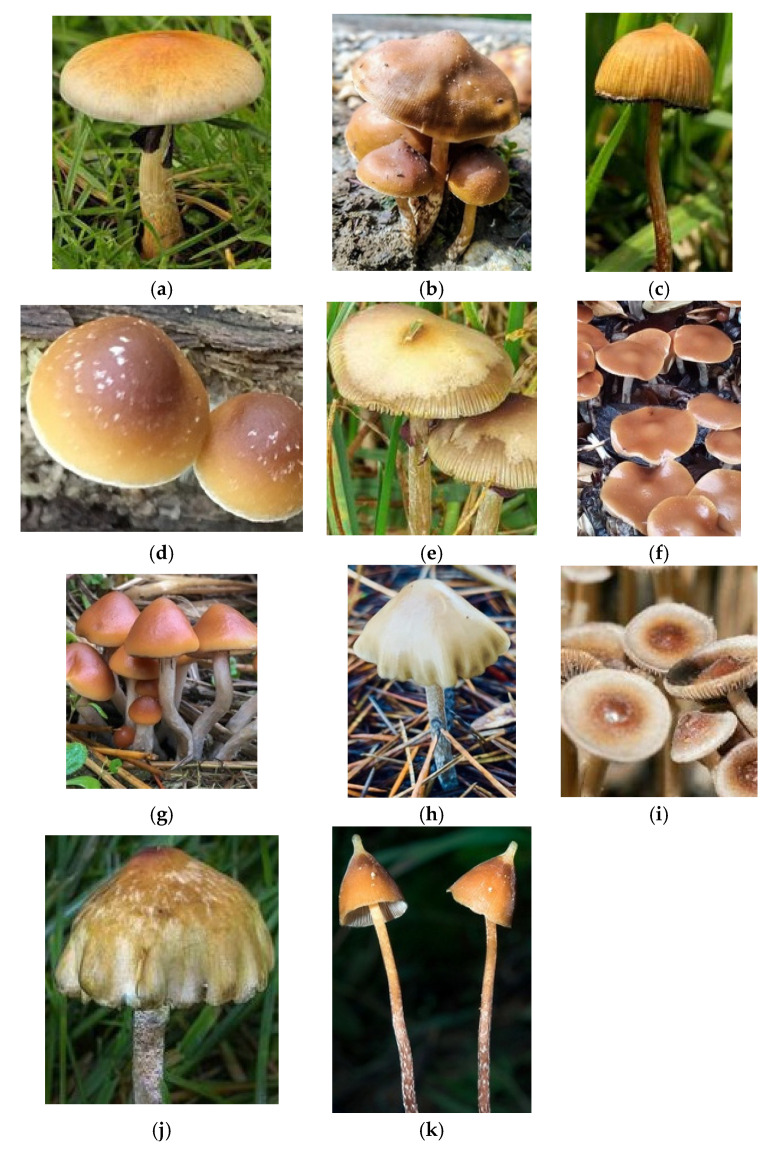
Examples of Magic mushrooms (psilocybin-producing mushrooms). (**a**) *Psilocybe cubensis* (Earle) Singer a.k.a *Stropharia cubensis* [131]. (**b**) *Psilocybe caerulescens* Murrill (a.k.a. Landslide Mushrooms, Derrumbes) [132]. (**c**) *Psilocybe mexicana* R. Heim (a.k.a. Teonanacatl, Pajaritos) [133]. (**d**) *Psilocybe caerulipes* (Peck) Sacc. (a.k.a Blue Foot Mushroom) [134]. (**e**) *Psilocybe stuntzii* Guzmán and J. Ott (a.k.a. Blue Ringer Mushroom, Stuntz’s Blue Legs) [135]. (**f**) *Psilocybe cyanescens* Wakef. (a.k.a. Wavy Caps) [136]. (**g**) *Psilocybe azurescens* Stamets and Gartz (a.k.a Flying Saucer Mushrooms)’ [137]. (**h**) *Psilocybe pelliculosa* (A.H. Sm.) Singer and A.H. Sm. [138]. (**i**) *Psilocybe tampanensis* Guzmán and Pollock (a.k.a Magic Truffles, Philosopher’s Stone [139]. (**j**) *Psilocybe baeocystis* Singer and A.H. Sm. [140]. (**k**) *Psilocybe Hoogshagenii R. Heim nom. inval*. (a.k.a. Little Birds of the Woods) [141].

Refer to Figure 2 for the historical timeline of psychedelic drugs and Figure 3a–m for examples of magic mushrooms (psilocybin-producing mushrooms). Over 100 species of mushrooms of the genus *Psilocybe* produce psilocybin [2,107].

The John Hopkins Psychedelic Research Unit claims recognition for being the first to research psilocybin since the 1970s [108]. Between 2015 and 2020, nearly 550 grants have been awarded to research institutes engaged in psychedelic research [109]. In modern-day research, the majority of classic psychedelic neurotherapeutics research is focused on psilocybin’s potential in mood and anxiety disorders such as cancer-related psychiatric distress [2]. Table 2 below lists major institutes and organizations involved in psychedelic research.

## 2. Examples of Psilocybin Producing Mushrooms

Psilocybin is produced by many species of mushrooms that are distributed globally [5]. These include countries such as the USA, S.E. Asia, Europe Mexico and Central America [15,16,65]. The *Psilocybe cubensis* mushroom is the most widespread species of the Psilocybe genus [15,16,63].

## 3. Psilocybin Synthesis

**Figure 4:**
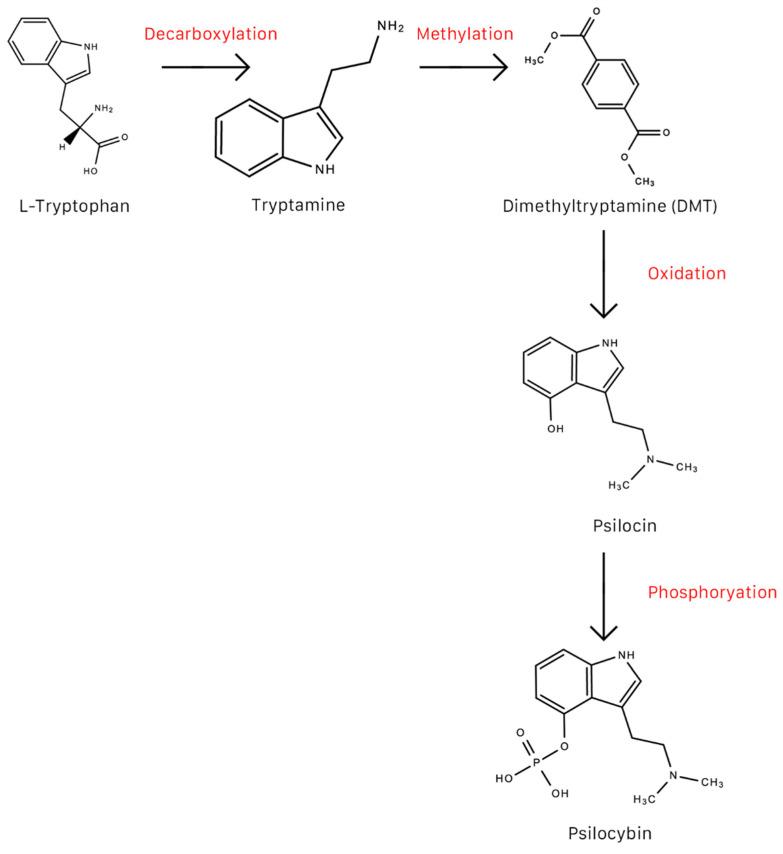
Conversion of L-tryptophan to psilocybin.

**Figure 5:**
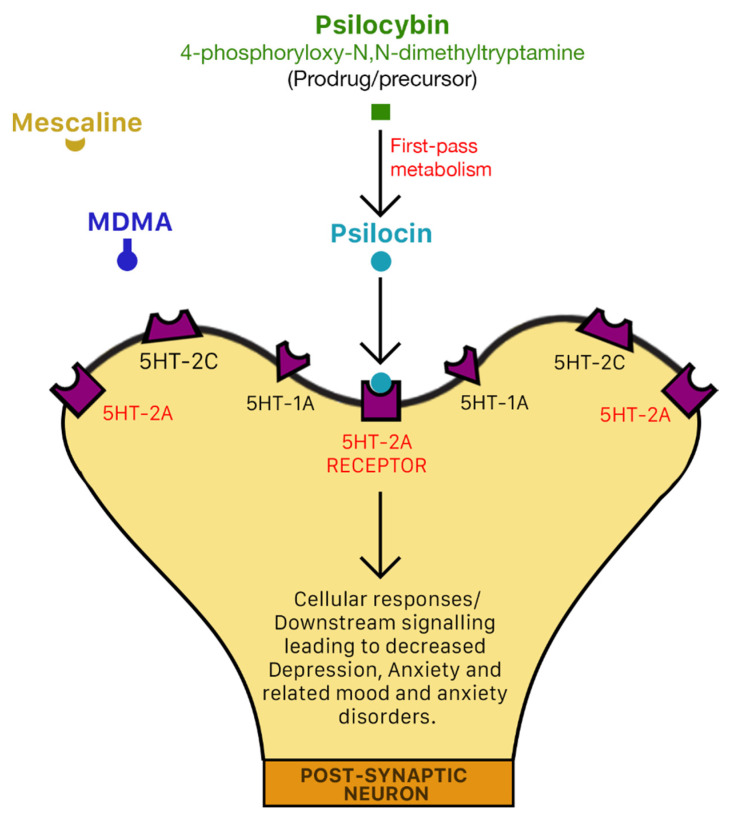
Mechanism of action of psilocin. Psilocybin binds with high affinity to 5-HT_2A_ [5]. 5-HT serotonin receptors are densely located in areas of the brain that are responsible for the mediation of mood and anxiety disorders such as the pre-frontal cortex. Molecular mechanisms of action have not yet been elucidated.

Psilocybin may be synthesized in a number of ways. Figure 4 is one example of how psilocybin may be converted from L-tryptophan. In humans, psilocybin is rapidly dephosphorylated to psilocin (4-hydroxy-*N,N*-dimethyltryptamine) by alkaline phosphatase in the liver [65,142] and nonspecific esterase in the intestinal mucosa [143]. In rodents, psilocybin is completely converted to psilocin before it enters systemic circulation [5] (Figure 5). It is psilocin that is the main pharmacologically active substance in magic mushrooms, not psilocybin [5,144], despite the common conception that it is psilocybin that produces the psychotomimetic effects. Psilocybin is considered a prodrug to psilocin [145].

### 3.1. Production of Synthetic Psilocybin

As a result of the increased need for psilocybin in recent years, due to renewed research focus, the market demands have to be met through synthetic psilocybin production [146]. Even though there is interest in the extraction of psilocybin from naturally growing or cultivated mushrooms, the psilocybin yield obtained (0.1–0.2% of dry weight) is not economically viable for drug research and development, and such may be limited to just recreational uses [143]. The observed variations in batches of psilocybin extracted from different sources further complicate the dependence on psilocybin directly extracted from mushrooms [146].

Most of the psilocybin that is produced synthetically is done through a complicated and expensive chemical synthesis as described by Nichols and Frescas in 1999 [147]. Although this method was an improvement from the initial method discovered by Hoffman and colleagues in 1958 [94], the final step focused on the psilocin phosphorylation to produce psilocybin, as well as the stereoselective 4-hydroxylation of the aromatic ring [148]. A newly patented method by COMPASS pathways increases the yield of semi-pure psilocybin to 75% (compared to 20% as was initially reported by Hoffman and colleagues in 1959) [149,150]. Despite yield increase, this method has proven expensive given the requirement for 4-hydroxyindole as the starting substrate, which is expensive and may result in high production costs [146].

The bioengineering of psilocybin has also been explored, which successfully can significantly reduce chemical synthetic costs through the production of psilocybin from cheaper start-up materials such as glucose. Following the elucidation of the biosynthetic pathway for the production of psilocybin in *P. Cubensis* in 2017 [151], bioengineering has led to the production of psilocybin in the filamentous fungi *Aspergillus nidulans,* with yields of up to 1.16 g/L [152]. Other methodologies have since been developed with an increased titer of 1.16 g/L, relying on the in vivo bioconversion of substrates 4-hydroxyindole, serine and methionine by *Escherichia coli* (*E. coli*) [153]. This method is currently not scalable as it relies on expensive startup substrates which can be used to produce psilocybin in cheaper chemical synthesis methodologies. A methodology for de novo production of psilocybin and tryptamine derivatives in *Saccharomyces cerevisiae,* utilizing knowledge of the psilocybin biosynthetic pathway elucidated in *P. cubensis* [154]. The pathway is supplemented with a novel cytochrome P450 reductase enzyme resulting in improved yields of 627 ± 140 mg/L of psilocybin and 580 ± 276 mg/L of psilocyn [154].

The production of psilocybin and psilocyn from *S. cerevisiae* is a significant achievement in developing cheaper psilocybin synthesis methodologies. Given the extensive use of *S. cerevisiae* industrially, as well as limited tryptophan derivatives produce, *S. cerevisiae* use for psilocybin production can significantly improve consistency in titer as well as optimize downstream processing [155].

Continuous research is needed nonetheless for the optimization of psilocybin synthesis. Given the upward trend in psilocybin research in recent years, the industry can only further benefit from optimized synthesis methodologies.

### 3.2. Mechanism of Action of Psilocybin

Psilocin reacts agonistically with serotonin (5-hydroxytryptamine) type 2A (5-HT_2A_) receptors to produce a “mystical-like” hallucinatory effect [5,99] (Figure 5) due to induced frontal hyper-frontality [5], which in turn mediates its anti-depressant and anti-anxiety effects [15,68]. One possible anti-depressant mechanism of action of psilocybin is via deactivation or normalization of the hyperactivity of the medial prefrontal cortex (mPFC) [156,157,158]. During depression, the mPFC is typically hyperactive [159].

Anti-depressant properties of psilocybin are mediated via modulation of the prefrontal and limbic brain regions, with the inclusion of the amygdala [160]. The amygdala plays an essential role in perception and emotion-processing networks [158]. In cases of depression, an individual typically loses responsiveness to emotional stimuli [44]. On the same tangent, it is also suggested that the hyper-frontal metabolic pattern produced after psilocybin administration and 5-HT_2A_ receptor activation is comparable to metabolic patterns produced in acute psychotic episodes in chronic schizophrenics [65].

It is also reported that psilocybin binds with high affinity to the 5-HT_2A_ serotonergic receptor subtype, but with low affinity to the 5-HT_1A_ serotonergic receptor subtype [5]. The interaction of psilocybin and psilocin with 5-HT_2A_ receptors to produce psychotomimetic effects has been confirmed in experiments with ketanserin, a 5-HT_2A_ antagonist that attenuates the effects of psilocybin [5,72]. In addition to interaction with 5-HT_2A_ receptors, it is also suggested that the psychopharmacological action of psilocybin may also be mediated by non-5HT2 receptors [7,143,158]. Psilocybin and psilocin also interact with the 5-HT_1D_ and 5-HT_2C_ receptor subtypes [5].

Psilocybin is reported to result in significant changes in brain dynamics and functional connectivity (FC) between areas of the brain [160,161,162,163]. Psilocybin-induced alteration in brain connectivity involves the disintegration of associative networks and integration of sensory function networks [65]. It is suggested that this dissociation may mediate the subjective effects of psilocybin use and a state of unconstrained cognition [65]. On the same tangent, a possible mechanism of action behind psilocybin’s psychotomimetic effects are via interactions with feedback loops between the cortex and thalamus [5]. Psilocybin administration produces general cortical activation [65]. This is confirmed by increased levels of the cerebral metabolic rate of glucose (CMRglu) in the prefrontal cortex, anterior cingulate, temporal cortex, and putamen [63]. This increase is positively correlated with hallucinatory “ego dissolution” [65]. The metabolic rate of glucose (MRGlu) also increased in distinct right-hemispheric frontotemporal cortical regions [118].

Serotonin 5-HT_2A_ receptors are distributed in multiple areas of the brain that play a role in psychosis and psychotic symptoms, such as the cerebral cortex (prefrontal cortex) and periphery [72], striatum, ventral tegmental area, and thalamus [164].

In addition to the presence of serotonergic cell bodies, dopaminergic cell bodies are also distributed in the VTA [165], an area of the brain that plays a role in reward-processed, and regulation of emotion and cognitive behaviours [166]. Although the neuropharmacological mechanisms of action of psilocybin are not definitively elucidated, there is evidence that, in addition to interaction with the serotonergic system, psilocybin also seems to interact, though not directly, with the mesolimbic dopaminergic pathway that plays a significant role in the brain’s reward system [165]. This proposed indirect mechanism of action is suggested by psilocybin’s low addictive/abuse potential [165]. On the same tangent, it has also been hypothesized that there is a positive correlation between depression and dopamine deficiency in the mesolimbic pathways [167].

It is also hypothesized that schizophrenia (and possibly other mood and anxiety disorders) is characterized by dysregulation/disbalance of serotonin and dopamine [168]. In acute psychoses, one study even concludes that 5-HT_2A_ and 5-HT_1A_ serotonergic receptors play an important role in the modulation of striatal dopamine release. This suggests that psilocybin may have significant potential in the treatment of schizophrenia and possibly other psychiatric disorders [168]. In another study, psilocybin was indirectly responsible for an increase in endogenous dopamine via a decrease in 11C-raclopride binding potential bilaterally in the caudate nucleus (19%) and putamen (20%) [169].

A 2021 study by Grandjean and colleagues investigated the effects of psilocybin on functional connectivity (FC) across the entire brain region in mice [170]. One possible mechanism of action by which psilocybin produces anti-depressant effects is via interaction with/alteration of the default-mode network (DMN) [171]. Using resting-state fMRI, psilocybin was shown to decrease functional connectivity within dopamine (DA)-associated striatal networks, in addition to demonstrating alteration (increase) of FC between 5-HT-associated networks and cortical areas [170]. This study confirms the interaction of psilocybin with the mesolimbic dopaminergic pathway to produce neural and psychological effects [170]. Data presented in another murine study suggest that psilocin, an active metabolite of psilocybin, has been shown to increase the concentrations of both extracellular dopamine and 5-HT in the mesoaccumbens and/or mesocortical pathway [172]. This presents yet another possible mechanism of anti-depressant action of psilocybin/psilocin, that is, the ability to increase dopamine, a neurotransmitter that is responsible for the regulation of emotions and even an individual’s physical well-being [173]. On the same tangent, concentrations of both extracellular dopamine and 5-HT in the ventral tegmental area (VTA) were not affected [172]. This further suggests that, in addition to the ventral tegmental area (VTA), the brain’s reward circuity may also be influenced by other regions of the brain.

5-HT_2A_ activation and subsequent activation of postsynaptic α-amino-٣-hydroxy-٥-methyl-٤-isoxazole propionic acid (AMPA) receptors by psilocybin is associated with increased glutamate concentration. Glutamate is responsible for normal, healthy brain functioning [174].

Psilocybin treatment, in some cases with psychological support, also resulted in increased responsiveness to positive emotional stimuli in the right amygdala [44,175] and decreased/normalization of responsiveness to negative or neutral emotional stimuli [112,158,176,177,178,179]. Psilocybin was also shown to attenuate amygdala activation in response to threat-related visual stimuli [177] and reduced threat-induced modulation of the primary visual cortex by the amygdala [177]. The amygdala modulates the primary visual cortex via top-down connectivity [177].

In contrast, other selective serotonin reuptake inhibitors (SSRIs) may produce anti-depressant effects by attenuating the hyper-responsiveness of the amygdala to fearful emotional stimuli, thereby inhibiting negative emotions [44]. Hyperactivity of the amygdala to fearful emotional stimuli is typically characteristic of depression [180]. SSRIs mitigate this hyperactivity to emotional stimuli [180] whereas psilocybin is suggested to increase amygdala activation to positive emotional stimuli [180,181]. In another study, psilocybin treatment also reduced anhedonia [182].

Barrett and colleagues also suggest that psilocybin may even influence brain plasticity as confirmed by the persisting positive effect and increased amygdala response to positive emotional stimuli up to one month post psilocybin treatment [112].

Unlike indoleamine LSD and other hallucinogens that that bind to dopamine D2 receptors to produce the typical dopaminergic “psychotic” experiences, psilocybin and psilocin have no affinity for dopamine D2 receptors [5,99,183], despite the existence of a functional interaction between the serotoninergic and central dopaminergic systems [5]. This functional interaction between the serotoninergic and central dopaminergic systems has been demonstrated in experiments with haloperidol, a D2 receptor antagonist that attenuates the psychotomimetic effects of psilocybin [5].

A study by Carhart-Harris investigated the effects of psilocybin on cerebral blood flow (CBF), and blood-oxygen-level-dependent (BOLD) resting-state functional connectivity (RSFC) via functional magnetic resonance imaging (fMRI) [171]. Post psilocybin treatment, authors reported decreased amygdala CBF associated with reduced symptoms of depression, and increased resting-state functional connectivity within the default-mode network (DMN) [184], increased RSFC in the ventromedial prefrontal cortex-bilateral interior lateral parietal cortex, and decreased RSFC in the parahippocampal–prefrontal cortex [171]. Alteration of the default mode network is characteristic of mood and anxiety disorders [185] and another possible mechanism of action by which psilocybin produces anti-depressant effects is via interaction with the DMN [171], via disruption of functional connectivity between the medial temporal lobe (MTL) and the DMN [186].

Another fMRI study [181] reports a decreased functional connectivity between the amygdala and the ventromedial prefrontal cortex (vmPFC) in response to fearful and neutral (but not happy) faces after psilocybin treatment. The ventromedial prefrontal cortex (vmPFC) is responsible for emotional processing, action, cognitive behaviour and goal-orientation, and demonstrates top-down inhibitory control on the amygdala [181]. It is suggested then that psilocybin treating, which decreases functional connectivity between amygdala and the ventromedial prefrontal cortex (vmPFC) in response to fearful and neutral (but not happy) faces, also decreases the top-down inhibitory control that the vmPFC has on the amygdala, and ultimately results in increased amygdala activity [181]. In medication-naïve individuals, decreased functional connectivity between the amygdala and the left rostral prefrontal cortex (left rPFC) is characteristic of major depressive disorder [187]. 

Also characteristic of depression and schizophrenia is an alteration of serotonergic signalling [188]. Thus, drugs that target serotonergic receptors in the prefront cortex may be of clinical importance [188].

### 3.3. Effects of Magic Mushrooms

The effects of magic mushrooms are dependent on the species of mushroom (and ultimately the concentration of active metabolites in a given species), an individual’s mindset an individual’s body type (particularly weight, metabolism) and an individual’s level of tolerance. Psilocybin’s acute psychedelic effects typically become detectable approximately 30–60 min after low to moderate (2–10 g) dosing [41]. Another study reports a range of 3–5 mg p.o. to produce sympathomimetic effects, but not hallucinogenic effects [142]. Hallucinogenic effects are produced within a range of 8–25 mg within 70–90 min [142]. It has been demonstrated that equimolar amounts of psilocybin and psilocin produce similar psychotropic effects in humans [5,189].

Perceptible psychological effects of psilocin correlate with plasma levels between 4 ng/mL and 6 ng/mL [142]. Hasler and colleagues estimate the bioavailability of psilocin to 52.7% (after 10–20 mg psilocybin ingestion) [142]. After a rapid increase in plasma levels of psilocybin, followed by a plateau for approximately an hour, psilocybin levels wane significantly until barely detectable after 6 h [142].

Subjective effects may last between 3 and 6 h [5], after which effects subside to negligible levels [41]. The effects of psilocybin may be classified into four categories: (1) Perceptual, (2) Cognitive, (3) Emotional and (4) Ego Dissolution [99]. More simply, the effects of psilocybin use may be divided into psychic and somatic effects [5] (Table 3).

In clinical studies, the clinical outcome, and acute and long-term subjective effects of psilocybin administration are measured using questionnaires such as the Subjective experience (5D-ASC) [72], the Beck Depression Inventory (BDI) [72], the Profile of Mood States (POMS) [69], the State-Train Anxiety Inventory (STAI) [72], the Mystical Experience Questionnaire [72], and the Quick Inventory of Depressive Symptoms (QIDS) [72]. Table 4 lists additional acute and long-term, subjective effects of psilocybin administration. Table 5 lists participants’ first-hand experiences and subjective perspectives during and after psilocybin in a 2016 phenomenological study conducted by Zamaria and colleagues [15].

### 3.4. The Possible Entourage Effect Phenomena in Magic Mushrooms

The phenomenon of the entourage effect suggests that the sum of the contributing parts of a botanical or biological system produce a greater, synergistic effect in comparison to the effect of each individual part when presented alone. The entourage effect is mostly associated with *Cannabis sativa* L.

**Figure 6:**
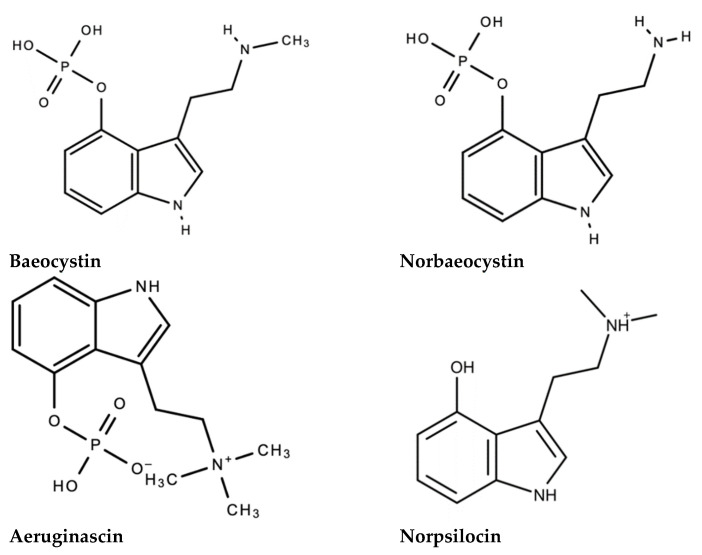
Chemical structures of recently discovered tryptamine derivates that may contribute/enhance the effects of psilocybin and psilocybin-assisted therapy.

Although psilocybin is the most popular and the most abundantly produced psychoactive compound/tryptamine derivative in magic mushrooms, other tryptamine derivatives such as psilocin [206], baeocystin [207], norbaeocystin [207], norpsilocin [207] (Figure 6) and the beta-carbolines such as harmane and harmol [208], may also enhance the effects of psilocybin and the efficacy of psilocybin treatment [208,209]. Thus, it is likely that these hallucinogenic compounds may work in tandem to produce a synergistic effect [208,210,211,212,213].

It should be noted that the ingestion of magic mushrooms, with multiple compounds present, will likely produce a different effect to the ingestion of a single, isolated compound, like pure psilocybin [208]. This is due to a difference between the pharmacology of whole magic mushrooms and a single, isolated pure compound [208]. This also suggests a synergism of multiple compounds in the mushroom.

In a marble-burying behavior study in mice that mimics anxiety and obsessive–compulsive disorder (OCD), Matsushima and colleagues report the findings that, at the same dose of 0.1 to 1.0 g/kg, a *psilocybe argentipes* mushroom extract was more effective than pure psilocybin alone at reducing marble-burying behaviour without affecting overall locomotion [213]. This too suggests the probable synergistic involvement of multiple bioactive active compounds in the mushroom extract [213].

In a species of magic mushrooms known as *Inocybe aeruginascens*, aeruginascin, a trimethylammonium analogue of psilocybin, is also produced in addition to psilocybin and baeocystin [214,215]. Aeruginascin demonstrates a high affinity for the 5-HT_1A_, 5-HT_2A_, and 5-HT_2B_ serotonin receptors [216] in producing euphoria-based hallucinogenic effects and, likely modulate the pharmacological action of psilocybin and the psilocybin-experience [217]. Norpsilocin is also a potent 5-HT_2A_ receptor agonist and is even reported to be more potent and possibly more efficacious than psilocin [207], while other the tryptamine derivatives baeocystin and norbaeocystin may serve as prodrugs to the bioactive compounds norpsilocin and 4-hydroxytryptamine, respectively [207], Baeocystin is a direct precursor to psilocybin [218], although it is not hallucinogenic by itself [207].

Further research is necessary to determine potential synergies amongst these compounds and other active and inactive molecules produced by magic mushrooms, and to determine the bioactivity of each compound on its own. On this tangent, practitioners should consider that some patients may require individualized treatments that may require a combined treatment approach as opposed to treatment with a single compound. Table 6 lists some factors that affect therapeutic/clinical outcome of psilocybin administration.

### 3.5. Adverse Effects, Risks and Contraindications

To reiterate, psilocybin is a Schedule I controlled substance as defined by the United Nations 1971 Convention on Psychotropic Substances. By this definition, psilocybin is considered to have a high abuse potential and is currently not accepted medically. In uncontrolled settings such as in recreation, abuse of psilocybin may lead to what is referred to as a “bad trip”. This is an undesired or even traumatic physical and emotional experiences characterized by altered visual perception, extreme distress, fear, lack of coordination, derealization, depersonalization, paraesthesiae, heightened fright, panic-attacks, traumatic flashbacks, paranoia, delirium, short-term psychosis and other symptomology characteristics of schizophrenia [228,229,230,231,232,233]. This undesired physical experience may also be accompanied by nausea, vomiting, mydriasis, headache, chills and drowsiness [229,230,234]. Some symptoms may even persist. A “bad trip” is typically treated with benzodiazepines [228].

Mushroom toxicity is also a risk associated with some species of psilocybin mushrooms. Though rare and typically accidental, mushroom poisoning is also a risk, and may lead to minor gastrointestinal illness (such as gastroenteritis), erythromelalgia, rhabdomyolysis, intestinal fibrosis, hypertension, hyperreflexia, liver failure, renal failure, convulsions, bradycardia, and tachycardia [228,230,235]. Mushroom poison may also require medical intervention or emergency hospitalization [230,231]. In general, alcohol and other drugs may exacerbate the psychological and physical risks of psilocybin abuse [229]. On the same tangent, individuals with a personal or family history of severe psychotic and psychiatric disorders are discouraged from using psilocybin, and by extension, other psychedelics [236].

In general, psilocybin is reported to have the most favourable safety profile of all psychedelic drugs [13,237,238]. Thousands of years of anecdotal evidence in addition to modern-day scientific studies confirm that psilocybin has low physiological toxicity, low abuse/addictive liability, safe psychological responses, no associated persisting adverse physiological or psychological effects during or after use [2,5,22,102,158,239,240]. Psilocybin overdose is very rare [241,242]. One such report of psilocybin overdose and subsequent fatality was specifically due to cardiac arrest, some 2–3 h after psilocybin ingestion, in a 24-year-old female who, 10 years prior, had a heart transplant due to end-stage rheumatic heart disease [243].

In patients with mental or psychiatric disorders, suicidal ideation and auto-mutilation are possible risks of magic mushroom ingestion and, though rare, have been documented in the literature [244]. Another risk is the possibility of exacerbating psychotic symptoms [192]. As a result, having psychotic disorders such as schizophrenic tendencies is a contraindication for undergoing psychedelic-assisted psychotherapy, particularly psilocybin-assisted psychotherapy [245].

It is also reported that repeated psilocybin use will build high tolerability but will not lead to physical dependence [5,158,246]. Cross-tolerance with other psychedelics such as LSD and mescaline [7,228,247]) is also a possibility. Discontinued psilocybin use does not typically cause adverse physical effects or symptomology related to drug withdrawal [228]. There is also a chance that psychological withdrawal may occur [228].

The Registry of Toxic Effects of Chemical Substances (RTECS) has assigned psilocybin a therapeutic index of 641, associated with a relatively better safety profile in comparison to nicotine and aspirin, with values of 21 and 199, respectively [102]. Essentially, this means that psilocybin has very low chronic toxicity, moderate acute toxicity, negligible public health and criminal effects [233]. To date, there is no standard value for a lethal dose at neither the recreational nor medicinal levels in humans [240].

Risks associated with psilocybin may be prevented or alleviated with the implementation of a medically supervised setting, professor preparatory counseling to induce the right patient mindset, and adequate professional clinical psychological and physiological support [227]. Due to the low physiological toxicity, low abuse/addictive liability, safe psychological responses, no associated persisting adverse physiological or psychological effects during or after use, it is hypothesized that the lethal dose of psilocybin is far greater than the effective dose [248,249,250,251].

### 3.6. Pharmacokinetics of Psilocin

To reiterate, the effects of magic mushrooms are dependent on the species of mushroom (and ultimately the concentration of active metabolites in a given species), an individual’s mindset an individual’s body type (particularly weight, metabolism) and an individual’s level of tolerance.

Typically, individuals tend to feel the effects of psilocybin mushrooms anywhere between 10 to 40 min, peaks 60–90 min after ingestion of anywhere between 4–10 mg (an estimated 50–300 micrograms per kilogram (µg/kg) of body weight) and then subsides approximately six hours post-ingestion [119,233,241]. Recreationally, users typically ingest anywhere between 10–50 g of fresh mushrooms (1–5 g of dried mushrooms), which corresponds to a dosage of about 10–50 mg psilocybin [233]. According to a study by the John Hopkins University of Medicine, higher doses of psilocybin (20–30 mg/70 kg) directly correlate to positive persisting effects on behaviour, attitude, mood, and general outlook on life up to 14 months after follow-up [227]. On the same tangent, another study also suggests that an oral dose of 25 mg psilocybin (correlating to roughly 0.3 mg/kg of body weight) may be within the therapeutic window [252].

In another study, the pharmacokinetics of escalating oral doses of 0.3, 0.45, and 0.6 mg/kg in 12 healthy adults was also investigated [252]. Though psilocin clearance varied among patients (which may be due to varying rates of psilocin glucuronide metabolite hydrolysis across patients), a linear relationship was reported between psilocin clearance and the twofold range of doses [252]. The mean elimination half-life of psilocin was 3 h with a standard deviation of 1.1 [252]. Renal excretion accounted for less than the 2% intact psilocin found in urine [252]. No psilocybin was found in urine or plasma [252]. These results may mean that increasing dosages of psilocybin typically does not produce any serious physical or psychological effects [252].

**Figure 7:**
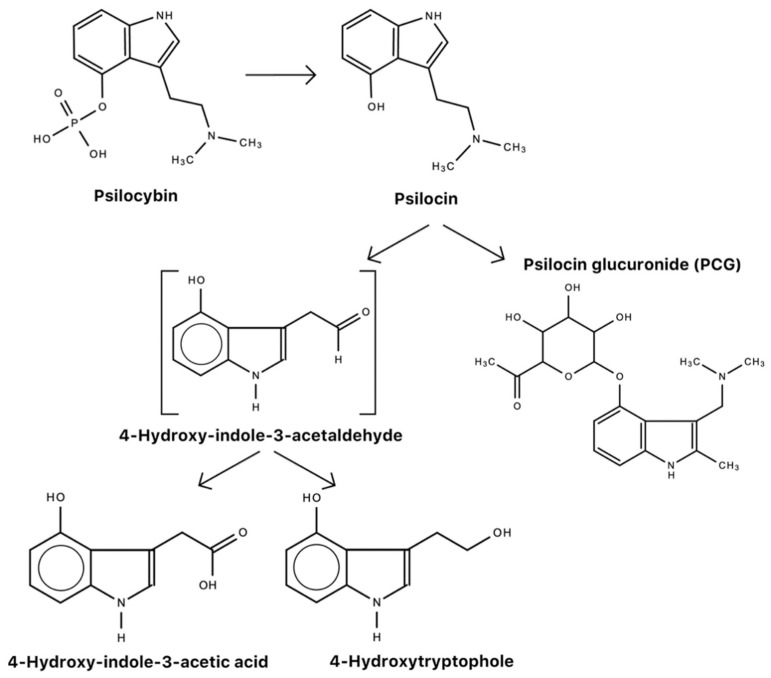
Metabolism of psilocybin [5,253,254].

Psilocybin has a shorter half-life and duration of action when given intravenously [5,142]. On the same tangent, psilocin has approximately two-thirds of unaltered (3–10%) psilocybin and glucoronidated metabolites are excreted through the kidneys after approximately 3 h [5]. Because the oral bioavailability of psilocin is 52.7% (after 10–20 mg psilocybin ingestion) [142], as a result, it is typically administered orally, but may also be administered intravenously, with comparable efficacy. Table 7 is a list of pharmacokinetic data on psilocin. Figure 7 shows the metabolic pathway of psilocybin.

After oral administration, most psilocybin, psilocin and glucoronidated metabolites are excreted via the kidneys, typically after about three hours [251]. After about 24 h, almost all psilocybin and psilocin are excreted from the body [251].

## 4. Economic Analysis of Neuropharmaceuticals Market

The increasing rate of global mood and anxiety disorders, particularly depression, the growing cultural and government acceptance, the increasing number of published scientific articles, and decriminalization, the burgeoning psychedelic industry has experienced an increase in economic value and a renewed pharmaceutical interest. This increase in value is also the result of more psychedelic companies going public and an increase in investors coming on board, especially since the COVID-19 pandemic and the subsequent collapse of investable cannabis opportunities [255].

In the 2nd quarter of 2019, more than USD 320 million was invested into psychedelic neuropharmaceutical development [255], followed by an estimated USD 100 million investment into various psychedelic research and clinical trials in the 3rd quarter of 2019 [255]. Globally, the psychedelic therapeutic market is predicted to reach a valuation of $6.8 billion by 2027, from USD 2 billion in 2019, at a growth rate of 16.3% [114]. The neurogenic market including mental health drugs, therapeutic services, neurodegeneration drugs and cognitive enhancement was valued at USD 373 billion [255]. The FDA’s recent approval of SPRAVATO^®^, a ketamine analog developed by Johnson and Johnson for use in patients suffering from treatment-resistant depression, and the approval of “breakthrough therapy” statuses for psilocybin treatments for Major Depressive Disorder (MDD) by Compass Pathways Ltd. and the Usona Institute, is also expected to spur the growth of psychedelic research.

Recently, Numinus Wellness Inc. was granted the first Health Canada license to produce and extract psilocybin from mushrooms [256]. This hallmark achievement will spur the growth of the synthetic psilocybin market in particular, and will allow for the rigorous scientific investigation of synthetic psilocybin as an alternative therapeutic option to naturally-occurring psilocybin. This achievement will also allow for the wide-scale production of naturally occurring psilocybin and standardization of cultivation, extraction and testing methodologies—and innovation in said technologies, thereof.

## 5. Conclusions and Future Direction

Psychedelic-assisted therapies may provide new and significant opportunities to current issues in the conventional treatment of psychiatric disorders. Psilocybin-assisted treatment may be feasible, efficacious, toxicologically safe, physiologically well-tolerated, and may have enormous potential in psychiatric medicine, as evidenced by decades of multiple clinical studies and thousands of years of anecdotal reports [2,5,158]. However, there are limitations that must be overcome before it can become an established part of psychiatric treatment. These limitations include the highly sensationalized global history and lingering negative stigmatization of psychedelic drugs, particularly in the United States, challenges with federal regulations, U.S. Food and Drug Administration (USFDA) and (European Medicines Agency) EMA approval and federal funding for clinical psychedelic studies [2], the lack of standardized psychedelic/psilocybin diagnostic and therapeutic practices [257], particularly in inducing “mystical experiences” that are essential to the outcome of psilocybin therapy [192], lack of larger, more sufficient double-blinded, randomized, clinical studies to assess safety, pharmacology and dose–response relationships for each mood and anxiety disorder [158], and challenges with intellectual property (IP) and securing enforceable patents, seeing that mushrooms grow naturally. A result of this IP challenge is the unwillingness of some investors to invest in the psilocybin industry. However, with the COVID-19 pandemic and the subsequent collapse of investable cannabis opportunities, other investors have found this to be the prime time to invest in psychedelics.

As magic mushrooms go mainstream again, multiple U.S. jurisdictions are pushing for the decriminalization of psilocybin mushrooms. In 2019, Denver (Colorado) and Oakland (California) were the first cities to decriminalize (though not legalize) psilocybin and plant and fungi psychedelics such as magic mushrooms, respectively [258]. In 2020, Washington, D.C., voted to decriminalize a select few plant and fungi psychedelics such as mescaline and psilocybin mushrooms [258,259,260,261]. In 2020, Oregon became the first state to legalize (and decriminalize) psilocybin mushrooms for personal development [261,262]. Other states like California are putting in grassroots efforts to decriminalize psychedelic mushrooms. Despite the efforts of multiple cities and states in North America in decriminalizing psychedelic mushrooms, only until there are changes in the federal regulatory framework and accessibility to federal funding will the psychedelic market reach its full potential.

On the contrary, in Jamaica, psilocybin mushrooms are legal and decriminalized, and there is a budding medical psychedelic tourism industry with the rise of psychedelic retreats like the Atman and Mycomeditations retreats [263]. As a result of Jamaica’s flexible regulatory framework for psychedelics, overseas companies are coming to the island to set up production and distribution infrastructure for psychedelic mushrooms [264]. In the future, we can expect the rise of psychedelic retreats and ultimately, medical psychedelic tourism in Jamaica.

Psychedelic research is also on the rise in Jamaica. In an attempt to convince the WHO to reschedule psilocybin out of Schedule 1 to more favourable scheduling, it is important that Jamaica’s growing psychedelic mushroom industry focus on and be driven by scientific research and evidence-based data.

Pioneering plant-based psychedelic research companies such as The Psyence Group, based in Canada, has partnered with the Biotech Research and Development Institute (BRDI) (University of the West Indies, Jamaica), to bring to market novel psilocybin treatments for mental health disorders in Jamaica. Psyence was one of the first to build and operate a federally licensed commercial psilocybin cultivation and production facility in the world [130]. Other overseas companies have also partnered with the University of the West Indies (UWI), Mona to conduct psychedelic research and psilocybin clinical trials studying Major Depressive Disorder and Addiction in Jamaica [264].

With an estimated 20% of Jamaicans suffering from anxiety and depression now exacerbated by COVID-19, psilocybin-assisted therapy may be a viable solution [264].

Additional limitations to psilocybin pharmaceutical research, and psychedelic research in general, include lack of data on psilocybin–drug interactions and combination-medicine studies [99], the lack of data on the effects of psilocybin on brain activity/dynamics/structure and neuroplasticity [99], and the lack of data on the molecular, neurobiological and psychological mechanisms of action and behavioural effects of psilocybin for each diseased state, and the mechanism of action behind persisting positive effects after psilocybin treatment.

The future of psilocybin-based neuropharmaceuticals may also involve the general research and development of psilocybin drugs, the development of individualized neuropharmaceuticals to meet the specific needs of a given patient, combination therapy of psilocybin or psilocin with other drugs (such as cannabis/cannabidiol) [265,266], conventional psychotherapy, and of non-psychoactive analogues of psilocybin [2]. It would also be interesting to study the synergistic effects of psilocybin in combination with other mind-altering and non-mind-altering drugs in the treatment of mood and anxiety disorders. Another interesting area study is the investigation of the possible potentiation of psilocybin’s chemical effect by the rituals that often accompany shaman-led sessions, although conversely, this could scientifically be viewed as a confounding variable.

## Figures and Tables

**Table 2:** Major institutes and organizations involved in psilocybin research.

	Institute or Organization	Some Publications/Areas of Study/Clinical Trials	References to Psilocybin Studies.
1.	Center for Psychedelic and Consciousness Research (John Hopkins University, USA) Founded in 2019.	Psilocybin can occasion mystical experiences having substantial and sustained personal meaning and spiritual significance. Survey of subjective “God encounter experiences”: Comparisons among naturally occurring experiences and those occasioned by the classic psychedelics; psilocybin, LSD, ayahuasca, or DMT Effects of psilocybin-assisted therapy for major depressive disorder: A randomized clinical trial. Psilocybin acutely alters the functional connectivity of the claustrum with brain networks that support perception, memory, and attention. Subjective features of the psilocybin experience that may account for its self-administration by humans: a double-blind comparison of psilocybin and dextromethorphan. Optimal dosing for psilocybin pharmacotherapy: Considering weight-adjusted and fixed dosing approaches.	[45,103,108,110,111,112,113,114,115]
2.	Department of Psychiatry (Harbor-UCLA Medical Center, USA).	Pilot Study of Psilocybin Treatment for Anxiety in Patients with Advanced-Stage Cancer	[33]
3.	Usona Institute (Wisconsin, USA).	2018—U.S. Food and Drug Administration (USFDA) approval for a psilocybin treatment for major depressive disorder (MDD)	[116]
4.	Compass Pathways Ltd. (London, UK).	2018—U.S. Food and Drug Administration (USFDA) approval of “breakthrough therapy” status in 2018 for a psilocybin treatment they developed for treatment-resistant depression	[104]
5.	Cybin, Corp. (Toronto, ON, Canada)Founded in 2019.	A psilocybin drug targeting MDD (in phase 2a and phase 2b of clinical trial). A phase 2 clinical trial investigating the delivery of psilocybin through an oral film. A study into a transdermal, “slow-dose” psilocybin delivery mechanism. A study on clinical safety and efficacy of targeting micro-dosing anxiety, ADHD and overall cognitive flexibility.	[117,118,119]
6.	Multidisciplinary Association for Psychedelic Studies (MAPS). Founded in 1986.	v. Positron emission tomography and fluorodeoxyglucose studies of metabolic hyper-frontality and psychopathology in the psilocybin model of psychosis. vi. Neurometabolic effects of psilocybin, 3,4-methylenedioxyethyl-amphetamine (MDE) and d-methamphetamine in healthy volunteers. A double-blind, placebo-controlled PET study with [18F] FDG. vii. The pharmacology of psilocybin. viii. Acute psychological and physiological effects of psilocybin in healthy humans: a double-blind, placebo-controlled dose-effect study. Psilocybin can occasion mystical-type experiences having substantial and sustained personal meaning and spiritual significance Response of cluster headache to psilocybin and LSD. Psilocybin links binocular rivalry switch rate to attention and subjective arousal levels in humans. Mystical-type experiences occasioned by psilocybin mediate the attribution of personal meaning and spiritual significance 14 months later Novel psychopharmaceutical therapies for psychiatric disorders: psilocybin and MDMA	[5,47,68,108,117,118,119,120,121,122]
7.	Harvard Psilocybin Project (Department of Psychology at Harvard University, USA).	Harvard-Concord Prison Experiment (1961-1963) studying the effects of psilocybin-assisted psychotherapy on rates of recidivism and the effects of consciousness-expanding drugs on prisoner rehabilitation. Reactions to psilocybin administered in a supportive environment. A new behavior change program using psilocybin.	[97,98,123]
8.	Heffter Research Institute (founded and Incorporated in New Mexico, USA 1993).	Pilot study of the 5-HT2AR agonist psilocybin in the treatment of tobacco addiction. Psilocybin-assisted treatment for alcohol dependence: a proof-of-concept study. Rapid and sustained symptom reduction following psilocybin treatment for anxiety and depression in patients with life-threatening cancer: a randomized controlled trial. Psilocybin-assisted group therapy for demoralized older long-term AIDS survivor men: An open-label safety and feasibility pilot study Safety, tolerability and efficacy of psilocybin in 9 patients with Obsessive–Compulsive Disorder.	[23,28,32,35,53]
9.	University of New Mexico Health Sciences Center (USA) in association with the Heffter Research Institute and University of New Mexico.	A Double-Blind Trial of Psilocybin-Assisted Treatment of Alcohol Dependence	[23]
10.	Department of Psychiatry (Yale University, USA).	The role of psychedelics in palliative care reconsidered: A case for psilocybin The Yale Manual for Psilocybin-Assisted Therapy of Depression (using Acceptance and Commitment Therapy as a Therapeutic Frame) Clinical trial: Psilocybin for the Treatment of Cluster Headache; Safety and Efficacy of Psilocybin for the Treatment of Headache Disorders Psilocybin—Induced Neuroplasticity in the Treatment of Major Depressive Disorder.	[124,125,126,127]
11.	Canadian Centre for Psychedelic Science.	Micro-dosing as a response to the meaning crisis: a qualitative analysis. Micro-dosing Psychedelics: Subjective Benefits and Challenges, Substance Testing Behavior, and the Relevance of Intention.	[128,129]
12.	The Psyence Group (Toronto, ON, Canada).Founded in 1994.	Granted one of the first federally (Lesotho, Africa) licensed producers of medicinal-grade mushrooms for treatment of psychological trauma and its mental health consequences.	[130]

**Table 3:** Psychic vs. somatic effects of psilocybin.

Psychic Effects in Humans and Animals (in Medium Dose (12–20 mg p.o.)	Somatic Effects in Humans (Barely Noticeable/Secondary Pharmacological Effects) [5]
Stimulation of affect/affective activation [5], Hypnagogic experience [5], Dreams [5], Enhanced ability for introspection [5], Mystical-type experience, which predicted the success of the therapy and likelihood of persisting positive benefits [9,15], Illusions [5], Synaesthesia [5], and Alterations of thought and time sense [5],	At 8–12 mg p.o., i.m.; Mydriasis [189], Accelerated heart frequency [189], Slowed heart frequency [189], Hypotension [189], Hypertension [189], Nausea [189], Increased reflex tendineae [189], Decreased reflex tendineae [189], Dysmetria [189], and Tremors [189] At 0.11 mg/kg p.o, similar effects were observed in another study [190]. At 1.5 mg increased to 25 mg p.o. in three doses per day, for 21 consecutive days, another study reported no significant aberrations in the parameters above [191].

**Table 4:** Additional acute and long-term subjective effects of psilocybin administration.

	Effect	Reference
1.	Positive changes in personality and increased altruism. This may, in turn, have wider benefits to society and the global environment.	[192,193,194,195,196]
2.	Enhanced feelings of connectedness	[192]
3.	Enhanced-nature relatedness	[40,192]
4.	Pro-environmental behaviour	[192,197]
5.	Decreased violent and criminal behaviour	[198,199,200]
6.	Reduced suicidal ideation	[13,192,200]
7.	Protection against suicidality and psychological distress (lifetime psilocybin-use)	[13]
8.	Tempered politically authoritarian views	[40,192]
9.	Increase in personality domain of openness	[192,201,202]
10.	Ego dissolution. Reduction of egotistical attitudes, narcissism and induces greater prosocial behaviour.	[65,192,203]
11.	Sustained/persisting improvement in attitudes and behaviour.One study reports substantial decreases in depressive and anxious symptoms persisting up to 6 months after a single active treatment. In another study, participants report positive persisting effects in areas of mood, behaviour, and attitudes after up to 14 months after psilocybin therapy.	[2,15,204]
12.	Improved psychological flexibility and feelings of personal meaningfulness, and subsequent improved psychological outlook. Ability to reframe how a patient views their medical conditions, themselves, their lives and relationships with others.	[192]
13.	Increase in one’s subjective sense of wellbeing.	[121,158]
14.	Quantum change (meaningful personal transformations)	[192,203]
15.	Enhancement of “meaning responses”	[192]
16.	Increased meditation depth	[205]
17.	Increased incidence of positive self-dissolution	[205]

**Table 5:** A phenomenological study of participant’s experience and subjective perspective during and after psilocybin use report the following first-hand accounts [15].

	Subjective, First-Hand Accounts of Experience with Psilocybin Treatment	Reference
1.	- Transcendental experience - “Mystical” - “Divine” - “Otherworldly experience” - “Felt like I saw God” - “Deep spiritual experience” - “Connection to something spiritual” - “Connection and spirituality with God” - “Continuity and oneness with God” - “Bliss, heaven, nirvana” - “Mystical and transcendental experience akin to a deep or profound state of meditation or an egoless state” - “Amazement”	[15]
2.	Changes in outlook - “Major shift in attention and perspective of the world” - “Notable and intense change in perspective of the world” - “Noticeable intricacies in environment” - “Clarity” - “Inspiration for behavioral change” - “Reduction in being too concerned about things, in general. Not neglectful, but hopeful”. - “Acceptance of concern with aging and death”.	[15]
3.	- Unity consciousness and ego dissolution - “Perception of life as a deeper, richer experience” - “Greater connection to nature, to other people, and to all living things” - “At one with the universe and all of existence” - “Every particle of existence felt like an extension of myself” - “Increased “connectedness” and “acceptance”” - “Feeling of being able to explore oneself more with others” - “Intense, beautiful feeling of love and joy and gratitude for everything”	[15,196]
4.	Peace and Happiness - “Incredible, profound calmness and stillness” - “Happiness and contentment” - “Persistent happiness and joy”	[15]
5.	- Increased introspection - “Greater capacity to motivate and examine oneself” - “Greater understanding of self” - Self-assessment - “Looking at oneself with much more objectivity” - “Reception of thoughts from a wise place” - “Greater insight” - “Greater self-awareness” - “Greater ability to understand what will bring happiness” - “Greater sense of purpose and direction” - “Persisting feeling of self-awareness and insight” - “Greater trust in one’s feelings and experiences” - “Access to deeper parts of oneself” - “Greater connection to core values” - “Feeling more “grounded” - “Persisting insight” - “Awareness of emotions and thoughts and better ability to articulate one’s thoughts and emotions to others. - “Deep appreciation and sense of gratitude”	[15]
6.	Physical/Mental - Improvement of mental wellbeing - Increase in patience- Reduction in depressive thoughts - Reduction in anxiety - “Lighter, limber, more energetic” - Persistent reduction in psychological distress (anxiety, worry, and sadness) - “Expedited self-development” - “Healthy and beneficial behavioural changes” - “Ability to understand and deliberately divert focus from/mitigate depressive thoughts”	[15]

**Table 6:** Factors that affect therapeutic/clinical outcome of psilocybin administration.

	Factor	Reference
1.	Extra-pharmacological elements (as with any drug). These include age factors, body weight, body size, muscle mass, genetics, drug tolerance, drug interactions, drug purity, dosage, gender, recreational consumption and inexperience with recreational drug use, past experiences with drug use, mindset, setting (context in which drug is used), experimental setting, social interaction, cultural influences, medical history, placebo design and response to placebo, and drug instrumentalization (instruments used to administer drugs).	[192,205,219,220,221,222,223,224,225]
2.	Mindset - Patient attitude and expectations (psychological flexibility/ outlook) - Preconceptions of treatment - Affective processes	[2,192,221]
3.	Setting - Use of music and/or art - Use of religious and spiritual imagery - Engagement with nature - Use of a purpose-built facility	[192]
4.	Psychological support - Intensive clinical care/contact - Supportive and reassuring interaction with therapists/sitters. - Reliable induction of “mystical experience” by clinician/therapist. Mystical experiences after psilocybin administration directly correlate with therapeutic outcome and persisting positive subjective effects.	[15,192]
5.	Specific types of psychological experiences	[2]
6.	Treatment type - Some patients may require personalized/individualized treatment which may involve combination therapy with other drugs. Drug interactions may likely affect psilocybin treatment. - Treatments with mushroom extracts as opposed to pure isolated psilocybin may also have different outcomes.	
7.	Type of mood or anxiety disorder	
8.	Degree of suicidality (ideation and actual attempts) in a patient. It is recommended that such patients who will be less likely to benefit from such treatment should be excluded from psychedelic therapy.	[15,192]
9.	Patients with a family history of psychotic disorders. It is recommended that such patients who will be less likely to benefit from such treatment should be excluded from psychedelic therapy. Psychedelics may augment/compound symptoms of psychoses.	[192]
10.	Patients who score high on neuroticism, a Big Five higher-order personality trait. It is recommended that such patients who will be less likely to benefit from such treatment should be excluded from psychedelic therapy.	[192,226]
11.	Patients with history or current diagnosis of bipolar disorder and schizophrenia. It is recommended that such patients who will be less likely to benefit from such treatment should be excluded from psychedelic therapy.	[15,192]
12.	Patients at high risk for developing psychosis, even though psilocybin does not cause lasting anxiety, depression or psychosis. It is recommended that such patients who will be less likely to benefit from such treatment should be excluded from psychedelic therapy.	[192]
13.	Patients on other psychedelic/anti-psychotic/anti-depressant medications such as selective serotonin re-uptake inhibitors (SSRIs), haloperidol, tricyclic anti-depressants, lithium, and monoamine oxidase inhibitors.	[192,227]

**Table 7:** Basic pharmacokinetics of psilocin.

	Type of Administration	Mean Half-Life of Psilocin	Elimination Constant (k_C_)	Absorption Constant (k_a_)	References
1.	Mouth (PO)	135 min	0.307/h	1.307/f	[245]
2.	Mouth (PO)	163 min	-	-	[142]
3.	Intravenous	74 min	-	-	[142]

## Data Availability

Data sharing is not applicable to this article. No new data were created or analyzed in this study.

## Sample Availability

Samples of the compounds are not available from the authors.
